# Adrenal Hemangioma: Findings at Multidetector CT with Short Review of the Literature

**DOI:** 10.1155/2011/601803

**Published:** 2011-09-29

**Authors:** Mohamed Abou El-Ghar, Huda Refaie, Ahmed El-Hefnawy, Tarek El-Diasty

**Affiliations:** ^1^Radiology Department, Urology and Nephrology Center, Mansoura University, 35516 Mansoura, Egypt; ^2^Urology Department, Urology and Nephrology Center, Mansoura University, 35516 Mansoura, Egypt

## Abstract

We present the computed tomography (CT) imaging findings of a 44-year-old male with incidentally discovered right adrenal hemangioma displaying imaging pattern of nonadenomatous pattern, associated with multiple hepatic hemangiomata using 64-slice multidetector scanner with reviewing published CT imaging findings with short review of the literature.

## 1. Introduction

Adrenal gland cavernous haemangioma is an extremely rare benign vascular tumor composed of angioblastic cells that is mostly discovered incidentally [1]. The first report of adrenal cavernous hemangioma was published in 1955 [2], and only 56 cases have been reported to date. Its incidence have been increased in the last years due to the evolution of cross-sectional imaging including computed tomography (CT) and magnetic resonance imaging (MRI) with increased incidentally discovered silent adrenal lesions. We present the first report of image findings using 64-multidetector CT (MDCT) scanner of a case of multiple adrenal hemangioma and multiple hepatic hemangiomata. 

## 2. Case Report

A 44-year-old male with an incidentally discovered right adrenal mass with multiple hepatic focal lesions during routine ultrasound of the abdomen was examined. Multiphasic CT study was done on a 64-multislice helical CT scanner (Brilliance, Philips, The Netherlands) with a standard uniform protocol for all of our patients. We injected 120 mL of contrast material (Ultravist 300 (iopromide), Schering, Berlin) at a flow rate of 4 mL/sec using an automatic injector. The contrast was injected via the anticubital vein via a 19-gauge cannula.

The multiphasic CT study included a noncontrast scan of the upper abdomen from the diaphragm to the iliac crest, corticomedullary (CM) phase after 25 sec from injection of contrast for the kidney, then, after 10-sec delay after CM phase, we obtained nephrographic phase. Delayed CT scan was done after 10 minutes from contrast injection. 

The slice section for noncontrast and delayed phases was 5 mm with an overlap of 2.5 mm, and, for CM and nephrographic phases, was 2.5 mm with 1.25 mm overlap. 

**Figure 1:**
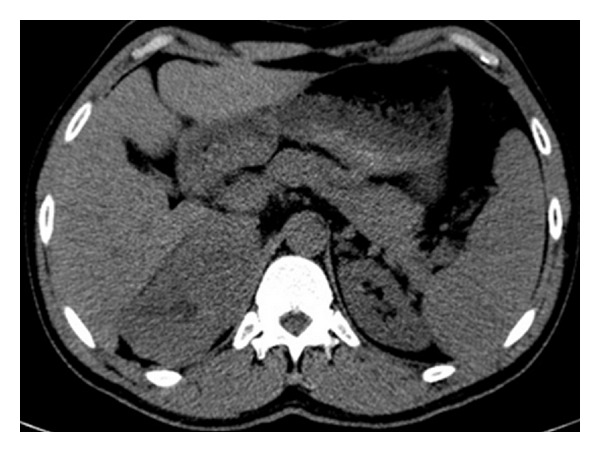
Axial noncontrast CT at the level of adrenal fossa shows a well-capsulated right adrenal solid lesion measured (11 × 6 cm) of relative isodense pattern in comparison to renal parenchyma with central hypodense area; the lesion displays 37 HU.

**Figure 2:**
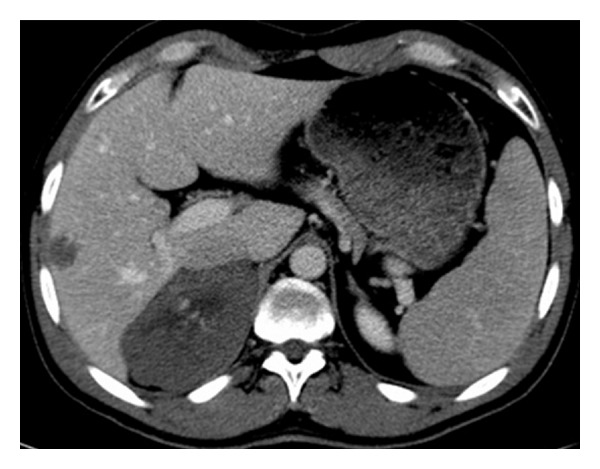
Axial postcontrast CT scan at CM phase shows a central patchy enhancement of the lesion displaying 160 HU, with nonenhancing peripheral part displaying 42 HU as well as the central hypodense area, there is a hypodense right hepatic focal lesion with nodular peripheral enhancement.

**Figure 3:**
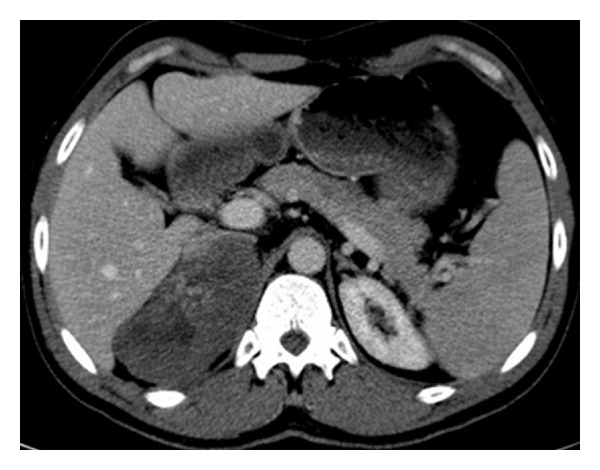
Axial CT scan at the nephrographic phase; there is still patchy central enhancement with nonenhancing peripheral part.

**Figure 4:**
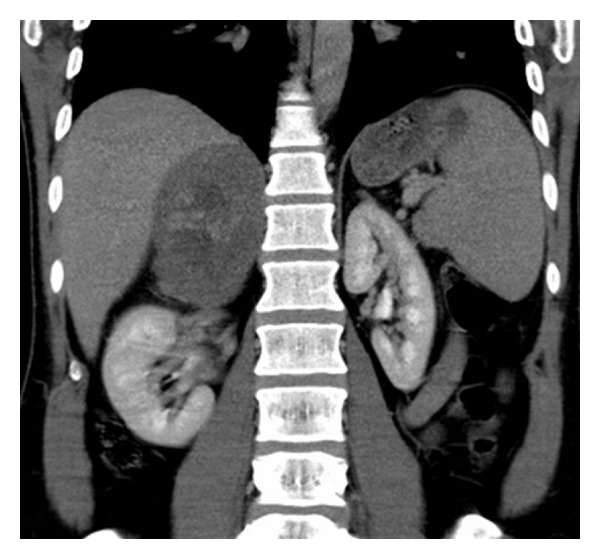
Coronal reformatted image at nephrographic phase shows the enhancing central area.

At noncontrast CT, there is a solid lesion measured (11 × 6 cm) of relative isodense pattern in comparison to renal parenchyma with central hypodense area occupying the right adrenal fossa and displaying 37 HU (Figure 1). At the CM phase, there was irregular patchy enhancement around the the nonenhancing central area displaying 160 HU, while the periphery of the lesion is nonenhancing (Figure 2). At the nephrographic phase, axial scan (Figure 3), and coronal reformat (Figure 4), there was more patchy central enhancement with nonenhancing peripheral part. At delayed scan, there is more or less homogenous enhancing with nonenhancing central scar tissue.

**Figure 5:**
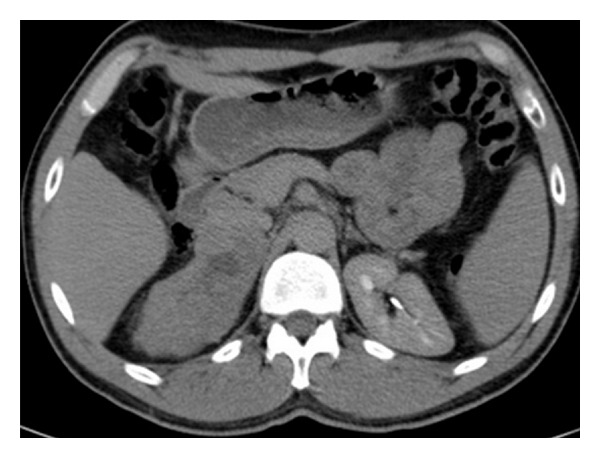
Axial CT at the delayed phase shows more or less homogenous enhancing with nonenhancing central scar tissue.

There were multiple hepatic hemangiomata at the liver with isodense pattern to blood vessels at noncontrast images and peripheral discontinuous ring enhancement at early postcontrast scans (Figure 2) and complete filling of the lesions at delayed images (Figure 5).

## 3. Discussion

The adrenal hemangioma is a rare benign tumor, but its incidence relatively increased in the last two decades due to widespread use of the modern cross-sectional imaging modalities. It poses problems in the differential diagnosis, because, preoperatively, they can be confused with adenomas or malignant tumors of the adrenal gland, and their preoperative diagnosis is very difficult [3]. It is most important that the cavernous hemangioma should be included in the differential diagnosis of adrenal neoplasm [4]. Cavernous adrenal hemangiomas are usually unilateral, become apparent in the sixth to seventh decade of life, with a 2 : 1 female-to-male predilection, and spontaneous life-threatening hemorrhage from adrenal hemangioma has been reported [5].

There were many reports of findings of central necrosis and/or fibrosis in the cases of adrenal hemangiomas [6–9]. Necrosis and fibrosis may be related to size: most reported adrenal hemangiomas have been greater than 10 cm in size [8]. In our case, the lesion measured 11 cm in its longest diameter with central necrosis that usually attributed to its large size and poor central vascularity. 

Reports of few cases who underwent conventional angiography showed peripheral pooling of the contrast, persisting well during the venous phase [10, 11].

The reported previous CT studies showed a characteristic peripheral patchy enhancement and highly dense peripheral rim. This pattern of peripheral spotty contrast enhancement with centripetal enhancement is crucial for diagnosing adrenal haemangioma [12], but all of cases are not imaged with multidetector scanners.

To our knowledge, our case is the first reported one imaged using 64-row multidetector scanner. The enhancing pattern was different than that reported by other scanners; in our case, the enhancement was seen central with peripheral filling of contrast and the central nonenhancing part looks as a hilum with enhancement started around it and extends peripherally. Also, we also discovered associated multiple hepatic hemangiomata with early peripheral nodular enhancement and central delayed filling. 

There are three cases of adrenal hemangiomas, coexisting with malignant tumors of other organs (nonsmall-cell lung cancer, common bile duct cancer, and gynaecological cancer), [13–15] have been reported in the literature.

The best way to determine the surgical indication is based on tumor size as determined by CT or MR imaging. Deckers et al. reported that it is safe to conservatively manage tumors smaller than 3.5 cm in size by regular follow-up imaging [16]. The lesion size and atypical pattern of enhancement in our case were the main challenge to excise the mass to exclude malignancy. The mass was removed via open surgery as the most reported cases with large masses were treated with open surgery [6, 17].
